# *GhSWEET42* Regulates Flowering Time under Long-Day Conditions in *Arabidopsis thaliana*

**DOI:** 10.3390/plants13162181

**Published:** 2024-08-06

**Authors:** Mengxue Du, Deying Wang, Jingyu Li, Taotao Zhu, Peng Lyu, Gang Li, Yi Ding, Xinxin Liu, Qingmei Men, Xiaofei Li, Yongwang Sun, Lingzhi Meng, Shangjing Guo

**Affiliations:** 1 School of Agricultural Science and Engineering, Liaocheng University, Liaocheng 252000, China; dumengxue1220@163.com (M.D.); wdy1999122@foxmail.com (D.W.); lijy0801@163.com (J.L.); 15208655400@163.com (T.Z.); m17663279431@163.com (P.L.); ligang_ice@163.com (G.L.); 15906337139@163.com (Y.D.); liuxinxin0518123@163.com (X.L.); 15163365595@163.com (Q.M.); lixiaofei9907@163.com (X.L.); sunyongwang@lcu.edu.cn (Y.S.); 2 School of Life Science, Qingdao Agricultural University, Qingdao 266000, China

**Keywords:** flowering time, long day, *GhSWEET42*, cotton, transcription factor, RNA-seq

## Abstract

Flowering in plants is pivotal for initiating and advancing reproductive processes, impacting regional adaptation and crop yield. Despite numerous cloned and identified flowering time genes, research in cotton remains sparse. This study identified *GhSWEET42* as a key determinant of the flowering time in cotton, demonstrating that its heterologous expression in *Arabidopsis* accelerated flowering under LD conditions compared to WT. Transgenic plants exhibited upregulated expression of the flowering inducers *AtFT*, *AtSOC1*, *AtGI*, and *AtFKF1*, alongside downregulated expression of the repressors *AtTSF*, *AtFLC*, and *AtRGL2*, correlating with the earlier flowering phenotype. *GhSWEET42* showed a constitutive expression pattern, with elevated levels in the leaves, petals, and flower buds, and was notably higher in early-maturing cotton varieties. Subcellular localization assays confirmed GhSWEET42’s presence on the cell membrane. Transcriptome analysis between WT and *GhSWEET42*-overexpressing *Arabidopsis* plants revealed 2393 differentially expressed genes (DEGs), spanning 221 biological processes, 93 molecular functions, and 37 cellular components according to Gene Ontology (GO) enrichment analysis. Kyoto Encyclopedia of Genes and Genomes (KEGG) pathway analysis categorized the DEGs into metabolism and environmental information processing. These findings enhance the understanding of *GhSWEET42*’s function and provide a foundation for elucidating the molecular mechanisms governing flowering time regulation in cotton.

## 1. Introduction

Flowering constitutes a vital phase in the life cycle of angiosperms, governing their reproductive success and seed development, and is intricately linked to crop yield and adaptability [1,2]. Premature flowering curtails resource accumulation by truncating the vegetative growth period, whereas delayed flowering heightens the risk of exposure to adverse environmental conditions [3]. Multiple endogenous genetic signals and external environmental factors influence flowering, with the photoperiod being a paramount environmental regulator [4]. Crops perceive photoperiodic conditions via an intrinsic biological clock in the leaves, transmitting signals to the shoot apex to trigger floral transition [3,5]. Based on photoperiodic flowering requirements, plants are categorized into short-day (SD), long-day (LD), and day-neutral types. *Arabidopsis thaliana*, a model organism, is a facultative LD plant, flowering earlier under LD conditions and later under SD conditions [3].

In *Arabidopsis*, extensive research has elucidated the molecular mechanisms regulating the flowering time, leading to the cloning and functional characterization of numerous related genes. Key players in this regulation include *CONSTANS* (*CO*), *FLOWERING LOCUS T* (*FT*), and *TWIN SISTER OF FT* (*TSF*) [6,7,8]. *CO* encodes a zinc finger protein that acts as a central regulatory factor in the photoperiod pathway, and its overexpression results in an early-flowering phenotype [9]. *SUPPRESSOR OF OVEREXPRESSION OF CONSTANS1* (*SOC1*) encodes a MADS-box protein, whose mutation counteracts the early-flowering phenotype induced by *CO* overexpression [10]. *FT* and *TERMINAL FLOWER 1* (*TFL1*) are pivotal regulators within the flowering pathway. *FT*, induced by *CO*, advances reproductive development and flowering, whereas *TFL1* inhibits flowering by downregulating downstream genes such as *LEAFY* (*LFY*) and *APETALA 1* (*AP1*) [11,12,13]. *TSF*, the closest homolog of *FT*, shares similar functions and is also regulated by *CO* and *FLOWERING LOCUS C* (*FLC*) [13,14,15].

Sugar is the primary energy source in plants, which necessitates efficient and timely transport for optimal growth and development [16]. Sugar transport proteins are essential for the translocation of sugars across cellular and tissue barriers [17,18]. SWEET (sugars will eventually be exported by transporters) proteins represent a significant class of sugar transporters, characterized by conserved MtN3_saliva transmembrane domains and diverse physiological roles [18,19]. In *Arabidopsis*, *AtSWEET8* is critical for exine pattern formation in microspores, with its loss of function severely compromising male fertility [19]. Knockout of *AtSWEET11* and *AtSWEET12* disrupts vascular development and freezing tolerance, while the *sweet11;12;15* triple mutant exhibits impaired embryo development, reduced seed weight, and lower starch and lipid contents [20,21]. In rice, *OsSWEET11* is highly expressed in pollen grains and inflorescence spikelets, with the *ossweet11* mutant showing decreased pollen starch content and impaired endosperm development [22,23]. *OsSWEET14* and *OsSWEET11* are crucial for grain filling, with double-knockout mutants significantly reducing the grain weight, yield, and grain-filling rate [23]. *GmSWEET15* facilitates sucrose transfer from endosperm to embryo during early soybean seed development, playing a pivotal role in embryo development [24]. The *ZmSWEET4c* mutant in maize demonstrates reduced starch content, grain weight, and embryo size compared to the wild type (WT), indicating its importance in seed filling [25]. Despite the critical roles of SWEET genes in plant growth and development, their involvement in flowering time regulation remains underexplored [18,20].

Cotton holds substantial economic value as a key cash crop [26]. Currently, two tetraploid cotton species, upland cotton (*Gossypium hirsutum* L.) and island cotton (*Gossypium barbadense* L.), along with two diploid species, Asian cotton (*Gossypium arboreum* L.) and grass cotton (*Gossypium herbaceum* L.), are widely cultivated [27]. *G. hirsutum* L. dominates global cotton fiber production, contributing over 95% [28]. The modern textile industry’s evolution has amplified the demand for natural fibers, necessitating the development of early-maturing cotton varieties to reduce harvesting times. The flowering time predominantly influences cotton plant maturation. Numerous genes associated with the flowering time in cotton have been identified in previous research. For instance, silencing *GhFUL2* elevates the expression levels of *GhFT* and *GhAP1.3*, prompting early flowering [29]. Transgenic plants with overexpressed *GhAP1-D3* exhibit earlier flowering while maintaining the yield and fiber quality [30]. In *Arabidopsis*, overexpression of *GhMADS42* and *GhAP1.7* triggers early flowering, whereas silencing *GhAP1.7* in cotton delays flowering [31]. *GhFPF1* overexpression in *Arabidopsis* induces early flowering by activating *AP1* and *FLC* expression [32]. GhGRF14, a GRF family protein, interacts with GhFT and GhFD to form florigen activation complexes that promote flowering, whereas GhGRF3, GhGRF6, GhGRF9, and GhGRF15 interact with GhFT and GhFD to form complexes that suppress flowering [33]. Despite the identification of several genes regulating flowering in cotton, the molecular mechanisms underlying their functions remain inadequately understood.

Previous investigations revealed that *GhSWEET42* influences the seed oil content and seed size [34]. This study explores a novel function of *GhSWEET42* in modulating the flowering time. In this study, a gene encoding the SWEET protein, *GhSWEET42*, from the cotton variety TM-1 was identified, and its protein structure and expression patterns were analyzed. Overexpression of *GhSWEET42* in *Arabidopsis* resulted in earlier flowering and an increased number of rosette leaves compared to the WT. Additionally, it influenced the expression of several flowering-related genes, including *AtFT*, *AtSOC1*, *AtGI*, *AtFKF1*, *AtTSF*, *AtFLC*, and *AtRGL2*. Transcriptome analysis identified 2393 DEGs between WT and *GhSWEET42* transgenic plants, with 1275 genes upregulated and 1118 downregulated, significantly enriched in 354 Gene Ontology (GO) terms and 12 Kyoto Encyclopedia of Genes and Genomes (KEGG) pathways. This research provides novel insights into the molecular mechanisms by which SWEET genes regulate the flowering time in cotton, offering valuable contributions to the development of early-maturing cotton varieties.

## 2. Results

### 2.1. Gene and Protein Sequence Analysis of GhSWEET42

**Figure 1:**
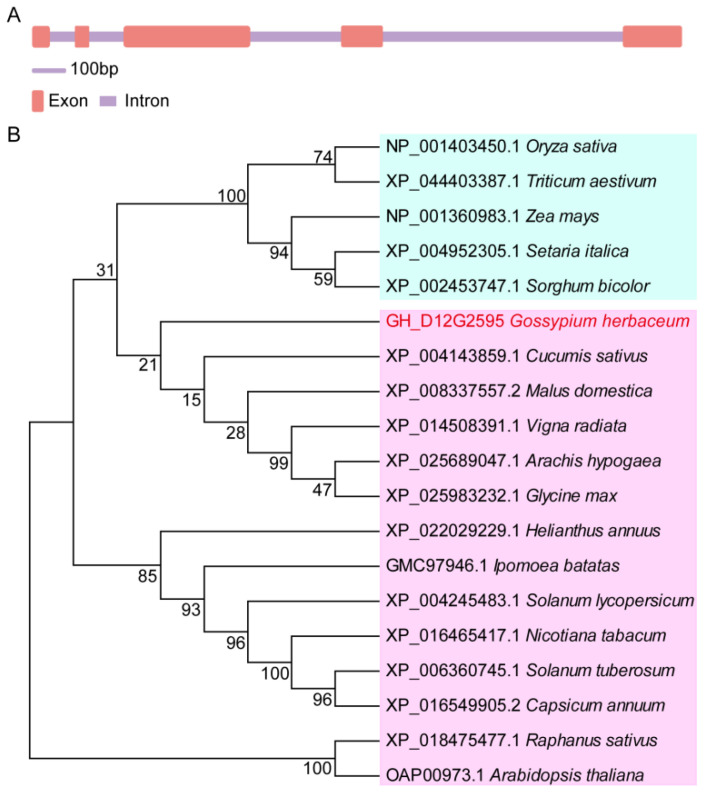
Gene structure and phylogenetic analyses of *GhSWEET42*. (**A**) The intron–exon structure of *GhSWEET42* is depicted, with exons represented by red lines and introns by purple lines. (**B**) Phylogenetic analysis of GhSWEET42, along with 18 homologous SWEET proteins in different species. GhSWEET42 is highlighted in red.

Prior research has identified the SWEET gene family as integral to plant growth and development. This study focuses on *GhSWEET42* (accession *GH_D12G2595*), a member of this gene family. The open reading frame (ORF) and genomic DNA fragment of *GhSWEET42* were amplified from the cotton variety TM-1. Sequence comparison between the genomic DNA and cDNA indicated that *GhSWEET42* comprises five exons and four introns (Figure 1A). The coding sequence (CDS) spans 765 nucleotides, encoding a 255 amino acid protein with a molecular mass of 28.18 kDa, an isoelectric point (pI) of 9.58, and seven conserved transmembrane domains (Figure S1A), consistent with the SWEET protein family [18,35].

Multiple sequence alignment of GhSWEET42 with homologous proteins from 18 species revealed high similarity with *Cucumis sativus* (69.76%), *Glycine max* (69.42%), *Ipomoea batatas* (68.1%), *Malus pumila* (67.07%), and *Vigna radiata* (66.67%), and comparatively lower similarity with *Solanum tuberosum* (64.64%), *Raphanus sativus* (60.91%), *Arabidopsis thaliana* (60%), *Nicotiana tabacum* (59.02%), and *Triticum aestivum* (58.7%) (Figure S1B). A phylogenetic tree was constructed to elucidate the evolutionary relationships of GhSWEET42 with proteins from other species, demonstrating that GhSWEET42 homologs segregate into two clusters: monocots and dicots. The closest homolog to cotton GhSWEET42 is from *Cucumis sativus* (XP_004143859.1) (Figure 1B).

### 2.2. Expression Analysis of GhSWEET42

**Figure 2:**
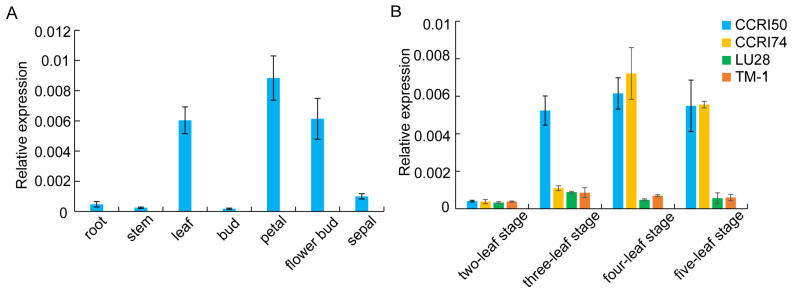
Expression profile of *GhSWEET42* in cotton. (**A**) *GhSWEET42* expression across various tissues. (**B**) *GhSWEET42* expression in leaves at distinct developmental stages. *GhHIS3* served as the internal control. Data are presented as mean ± SD (n = 3).

To delineate the tissue-specific expression profiles of *GhSWEET42* in cotton, the total RNA was isolated from various tissues, including the roots, stems, leaves, buds, flower buds, petals, and sepals. The expression levels were quantified via qRT-PCR. Notably, the highest expression was observed in the leaves, petals, and flower buds, whereas the roots, stems, buds, and sepals exhibited comparatively lower expression levels (Figure 2A).

To evaluate the *GhSWEET42* expression across various growth stages in different cotton varieties, its expression was examined in the leaves at four distinct stages across four varieties: two early maturing (CCRI50 and CCRI74) and two late maturing (Lu28 and TM-1). The *GhSWEET42* expression was lowest at the two-leaf stage in all the varieties, progressively rising to peak at the three-leaf stage in the late-maturing varieties and at the four-leaf stage in the early-maturing varieties, followed by a subsequent decline (Figure 2B). Moreover, the expression level of *GhSWEET42* in the early-maturing varieties was markedly higher than in the late-maturing varieties at both the four- and five-leaf stages (Figure 2B). This suggests that *GhSWEET42* is consistently expressed and significantly upregulated in the early-maturing varieties.

### 2.3. GhSWEET42 Is Localized to the Cell Membrane

**Figure 3:**
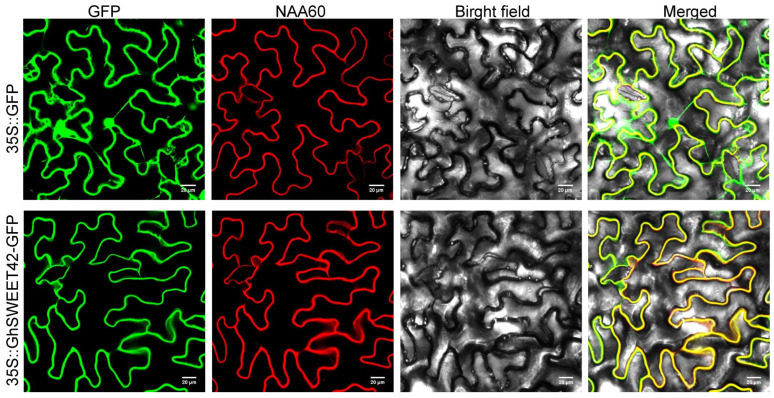
Subcellular localization of GhSWEET42 in tobacco leaves. Scale bar = 20 μm. GhSWEET42-GFP fusion protein localized in the plasma membrane. NAA60 is a cell membrane marker.

To predict the subcellular localization of GhSWEET42, the online tool Plant-mPloc was employed, indicating its localization to the plasma membrane (Figure 3). To corroborate this prediction, the full-length CDS of *GhSWEET42* was cloned into the N-terminus of the green fluorescent protein (GFP) within the pCAMBIA1305.1 vector, creating the GhSWEET42-GFP construct. This construct was then introduced into tobacco (*Nicotiana benthamiana*) leaves alongside the cell membrane marker NAA60 fused with mCherry. The co-localization of the GFP signals with the NAA60 red fluorescent signals confirmed the cell membrane localization of GhSWEET42.

### 2.4. Overexpression of GhSWEET42 Induces Early Flowering in Arabidopsis under LD Conditions

**Figure 4:**
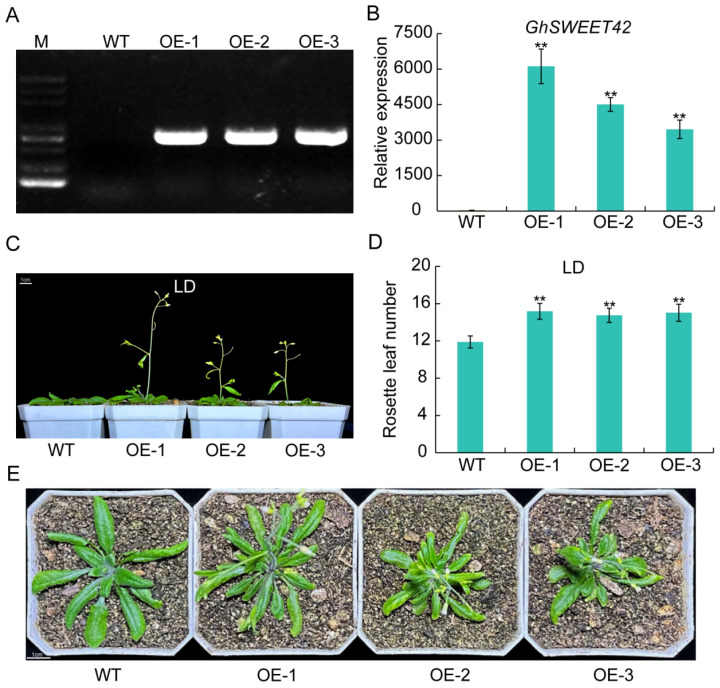
Overexpression of *GhSWEET42* in *Arabidopsis* under LD conditions. (**A**) PCR detection of *GhSWEET42*-transformed plants. (**B**) RT-qPCR analysis of *GhSWEET42* expression in the WT and transgenic *Arabidopsis* lines. (**C**) Phenotypes of the WT and *GhSWEET42*-OE lines under LD conditions. (**D**) Rosette leaf count in the WT and *GhSWEET42*-OE lines under LD conditions. (**E**) Rosette leaf size in the WT and *GhSWEET42* transgenic lines under LD conditions. Scale bar = 1 cm. *AtUBQ10* served as the internal control. Data are mean ± SD (n = 3). Significant differences indicated by asterisks at ** *p* < 0.01.

To elucidate the role of *GhSWEET42* in plants, the 35S::*GhSWEET42* overexpression vector was constructed and introduced into *Arabidopsis* (Figure 4A,B). Nine independent *GhSWEET42* transgenic lines were confirmed, and three homozygous lines with elevated *GhSWEET42* expression (OE-1, OE-2, and OE-3) were selected for detailed analysis. Phenotypic evaluation of the WT and *GhSWEET42*-overexpressing (*GhSWEET42*-OE) lines revealed that all the *GhSWEET42* transgenic *Arabidopsis* plants exhibited earlier flowering compared to the WT under LD conditions (Figure 4C) and produced more rosette leaves than the WT plants (Figure 4D). Under SD conditions, no significant differences in the flowering time or rosette leaf number were observed between the WT and transgenic lines (Figure S2A,B). Comparative analysis of the rosette leaf size at the onset of flowering under LD conditions showed that the *GhSWEET42*-OE plants had smaller rosettes compared to the WT (Figure 4E). These observations demonstrate that *GhSWEET42* accelerates flowering in *Arabidopsis* under LD conditions but not under SD conditions.

### 2.5. Expression Analysis of Flowering-Associated Genes in GhSWEET42 Transgenic Plants

**Figure 5:**
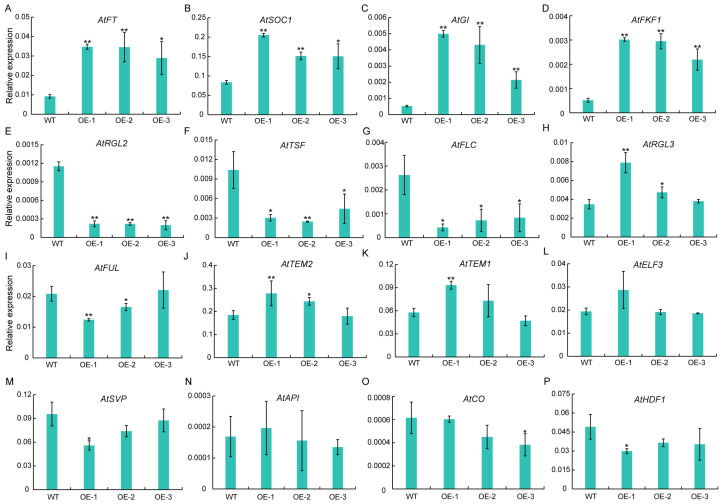
Expression levels of flowering-related genes in WT and *GhSWEET42*-OE plants. qRT-PCR assessed the transcript levels of 16 flowering-related genes in WT and *GhSWEET42*-OE seedlings at 28 DAG. Expression levels of *AtFT* (**A**), *AtSOC1* (**B**), *AtGI* (**C**), *AtFKF1* (**D**), *AtRGL2* (**E**), *AtTSF* (**F**), *AtFLC* (**G**), *AtRGL3* (**H**), *AtFUL* (**I**), *AtTEM2* (**J**), *AtTEM1* (**K**), *AtELF3* (**L**), *AtSVP* (**M**), *AtAPI* (**N**), *AtCO* (**O**), *AtHDF1* (**P**). *AtUBQ10* served as a reference. Data represent mean ± SD (n = 3). (* *p* < 0.05, ** *p* < 0.01).

To elucidate the regulatory interactions between *GhSWEET42* and various flowering-time regulators, the expression of 16 major flowering genes was quantified in the WT and *GhSWEET42*-OE lines under LD conditions. The *GhSWEET42*-OE lines exhibited a significant upregulation of the positive flowering regulators *AtFT*, *AtSOC1*, *AtGI*, and *AtFKF1* compared to the WT (Figure 5A–D). Conversely, the expression of the negative flowering regulators *AtTSF*, *AtFLC*, and *AtRGL2* was significantly downregulated in the *GhSWEET42*-OE lines relative to the WT (Figure 5E–G). No significant variation in the expression levels of *AtRGL3*, *AtFUL*, *AtTEM1*, *AtTEM2*, *AtELF3*, *AtSVP*, *AtAPI*, *AtCO*, and *AtHDF1* was observed between the *GhSWEET42*-OE lines and WT (Figure 5H–P). These data align with the early-flowering phenotype observed in the *GhSWEET42*-OE plants.

### 2.6. Transcriptome Analysis of the WT and GhSWEET42-OE Plants and DEG Identification

**Figure 6:**
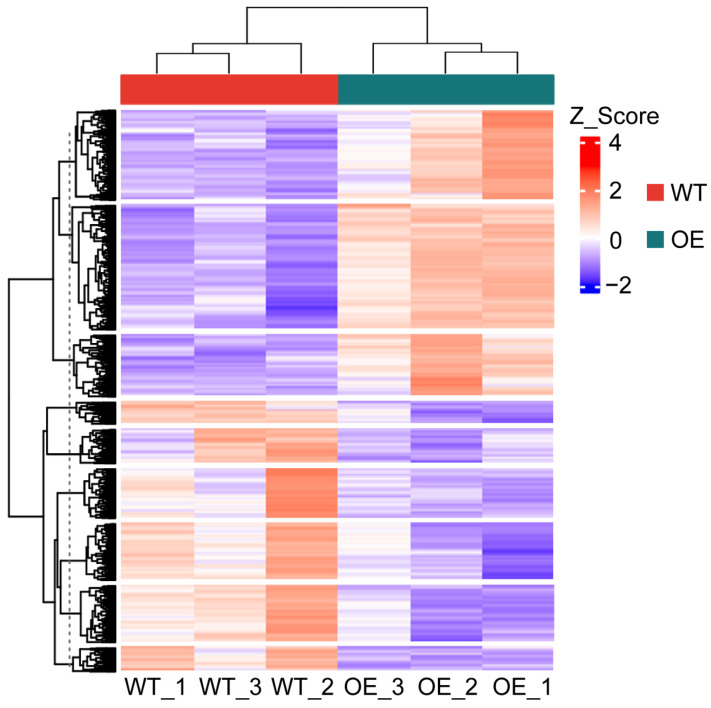
The bidirectional hierarchical clustering heat map illustrates the differentially expressed transcripts, with the genes displayed horizontally and one sample per column. Intensified red indicates higher gene expression levels, while intensified blue signifies lower expression levels.

To elucidate the regulatory mechanisms of *GhSWEET42*, a transcriptome analysis of WT and *GhSWEET42*-OE plants was performed using RNA-Seq with three biological replicates per genotype. Post-filtering, a total of 40.29 Gb of clean data were obtained, with each sample yielding an average of 6.71 Gb. Clean reads comprised over 99% of the total reads per sample, with the majority (>97%) uniquely aligning with the *Arabidopsis* reference genome (Table 1). The percentage of Q20 bases and Q30 bases exceeded 97% and 93%, respectively, with a GC content of at least 45% (Table 1). Bidirectional clustering analysis of the differentially expressed transcripts revealed significant differences between the WT and transgenic *Arabidopsis* (Figure 6). The high quality of the sequencing data supports its use in the subsequent analyses.

### 2.7. GO and KEGG Enrichment Analyses of the Identified DEGs

**Figure 7:**
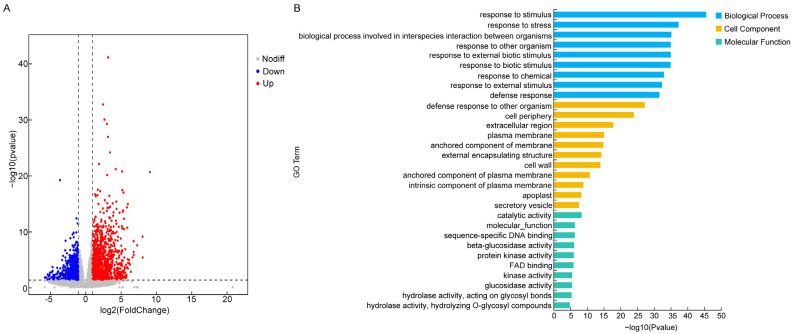
Comparative gene expression and GO enrichment analysis between the WT and transgenic lines. (**A**) The vertical dashed lines denote the differential expression fold change thresholds; the horizontal dashed line marks the significance level threshold. Red represents upregulated genes, blue denotes downregulated genes, and gray indicates non-significant differentially expressed genes. (**B**) GO terms for the DEGs of the WT and *GhSWEET42*-OE lines.

In transcriptomes from WT and *GhSWEET42*-OE plants, DEGs were identified based on the following criteria: |log2FoldChange| > 1, significant *p*-value < 0.05. Consequently, 2393 DEGs were identified, including 1275 upregulated and 1118 downregulated genes (Figure 7A, Table S1). GO enrichment analysis was conducted to elucidate the enriched categories among the DEGs (*p*-value ≤ 0.01), classifying the GO terms into three main categories: biological process (BP), molecular function (MF), and cellular component (CC) (Figure 7B, Table S2). In the BP category, 221 GO terms were identified, primarily encompassing the response to stimulus, response to stress, biological process involved in interspecies interaction between organisms, response to other organisms, response to external biotic stimulus, and response to biotic stimulus. For the CC category, the DEGs were predominantly associated with the defense response to other organism, cell periphery, extracellular region, plasma membrane, anchored membrane component, and external encapsulating structure terms. In the MF category, the enriched DEGs were linked to catalytic activity, molecular function, sequence-specific DNA binding, beta-glucosidase activity, protein kinase activity, and FAD binding (Figure 7B, Table S2). The GO classification analysis implies that the DEGs related to the flowering time in *Arabidopsis* are potentially involved in the responses to stimuli, cellular defenses, and catalytic reactions.

**Figure 8:**
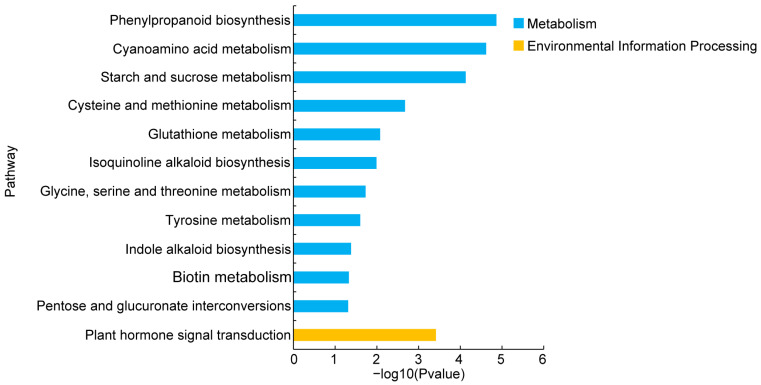
KEGG pathways enrichment analysis of the DEGs between the WT and *GhSWEET42*-OE plants.

Additionally, the KEGG pathway analysis elucidated the functions of the DEGs, identifying significantly enriched pathways (*p* ≤ 0.05) within two major categories: metabolism and environmental information processing (Figure 8, Table S3). The metabolic category encompassed pathways such as phenylpropanoid biosynthesis (ath00940, 27 DEGs), cyanoamino acid metabolism (ath00460, 18 DEGs), starch and sucrose metabolism (ath00500, 31 DEGs), cysteine and methionine metabolism (ath00270, 21 DEGs), glutathione metabolism (ath00480, 17 DEGs), indole alkaloid biosynthesis (ath00901, 4 DEGs), glycine, serine and threonine metabolism (ath00260,12 DEGs), tyrosine metabolism (ath00350, 8 DEGs), indole alkaloid biosynthesis (ath00901, 2 DEGs), biotin metabolism (ath00780, 4 DEGs), and pentose and glucuronate interconversions (ath00040, 15 DEGs). The environmental information-processing category included the plant hormone signal transduction pathway (ath04075, 43 DEGs) (Figure 8, Table S3). These data suggest that variations in the metabolic and environmental information-processing pathways contribute to the differences in the flowering time between WT and transgenic *Arabidopsis*.

### 2.8. Analysis and Annotation of Transcription Factors (TFs)

Differentially expressed TFs were identified among the DEGs and statistically analyzed, as depicted in Figure S3. A total of 195 TF-encoding genes were identified within the 2393 DEGs, representing 8.2% of the DEGs, with 120 genes upregulated and 75 genes downregulated. Among these, 34 TF families were identified (Figure S3), predominantly WRKY TFs (28 DEGs), NAC TFs (26 DEGs), ERF TFs (25 DEGs), MYB TFs (18 DEGs), and bHLH TFs (18 DEGs) (Table S4). This suggests that *GhSWEET42* may play a role in the transcriptional regulation of the flowering time.

## 3. Discussion

Upland cotton (*Gossypium hirsutum* L.) holds substantial economic value as a fiber and oil crop that is integral to global agricultural productivity. The flowering time, among various traits, plays a pivotal role in influencing the cotton yield. The flowering period of cotton is regulated by a short daylength, showing strict photoperiod sensitivity [36]. Over recent decades, research on flowering time genes in model plants like rice and *Arabidopsis* has elucidated the molecular mechanisms governing flowering. So far, only a few genes that regulate the flowering time in cotton have been identified, such as *GhFPF1* [32], *GhGASA14* [37], *GhAP1-D3* [30], and *GhAP1.7* [31]. SWEET proteins, functioning as sugar efflux transporters with the MtN3_saliva domain, orchestrate both reproductive and vegetative developmental processes [18,19]. A previous study showed that overexpression of *JcSWEET16* in *Arabidopsis* accelerates flowering [38]. In cotton, 55 putative SWEET proteins have been identified, although studies on these proteins are sparse [35].

In the present study, we identify a determinant of the flowering time, namely *GhSWEET42*, which negatively regulates flowering in *Arabidopsis* under LD conditions compared to the WT. Analysis of the *GhSWEET42* sequence and structure indicates that the protein has seven transmembrane domains (Figure S1A), consistent with the characteristic seven conserved MtN3_saliva transmembrane domains of SWEET proteins. *GhSWEET42* showed a constitutive expression pattern and exhibited higher expression levels in the leaves and floral organs, which is similar to *GhFPF1*, another flowering determinant, which is also highly expressed in flowers [32]. A previous study indicated that *GhAP1.7* expression was significantly elevated in early-maturing varieties compared to late-maturing ones. *GhSWEET42* showed a similar expression pattern to *GhAP1.7*. Our research confirms the role of *GhSWEET42* in regulating the flowering time [31].

We further analyzed the expression levels of 16 flowering-related genes in WT and *GhSWEET42* transgenic plants. *GhSWEET42* promoted flowering in *Arabidopsis* by upregulating the expression of flowering inducers such as *AtFT*, *AtSOC1*, *AtGI*, and *AtFKF1*, and by downregulating flowering repressors like *AtTSF*, *AtFLC*, and *AtRGL2*. This suggests that *GhSWEET42* may be involved in the vernalization pathway (*AtFLC*) [39], the autonomous pathway (*AtFT*, *AtSOC1*, and *AtFLC*) [11,40], the photoperiod pathway (*AtGI*, *AtFT*, *AtFKF1*, and *AtTSF*) [15,41,42,43], the gibberellin pathway (*AtRGL2*) [44] and the ambient temperature response (*AtFLC*) [45] (Figure 5A–G). Further studies involving genetic transformation in cotton are required to determine if *GhSWEET42* functions similarly in the regulation of the flowering time in cotton.

To elucidate the molecular regulatory mechanisms underlying *GhSWEET42*’s functions, a transcriptomic analysis was performed on WT and transgenic *Arabidopsis* plants at 21 days after germination (DAG), identifying 2393 DEGs, with 1275 genes upregulated and 1118 genes downregulated (Figure 7A, Table S1). The GO term analysis indicated that the identified DEGs were enriched across the three main GO categories: MF, BP, and CC, with “response to stimulus”, “cellular defense”, and “catalysis” being the most enriched terms in each category (Figure 7B, Table S2). The KEGG pathway analysis classified the DEGs into two primary categories: metabolism and environmental information processing. Notably, the metabolism category included pathways such as phenylpropanoid biosynthesis (ath00940, 27 DEGs), cyanoamino acid metabolism (ath00460, 18 DEGs), starch and sucrose metabolism (ath00500, 31 DEGs) and cysteine and methionine metabolism (ath00270, 21 DEGs), which were the most abundant in the metabolism category. These results indicate that *GhSWEET42* may regulate flowering through altering substantial metabolic processes. In the environmental information-processing category, 43 plant hormone signal transduction-related genes were identified (ath04075) (Figure 8, Table S3). Among these genes, 23 are upregulated and 20 are downregulated. Many of these genes have been previously confirmed to be involved in regulating the synthesis and signaling of auxins (*WES1*, *ERF1*, *BPN6*, *SAU36*, *LAX2*, *IAA6*, *SHY2*, *IAA7* and *IAA19*), jasmonic acid (JA; *JAZ10*, *JAZ25*, and *JAZ1*), gibberellins (GA; *RGL1* and *RGL2*), ethylene (*ERF2*), salicylic acid (SA; *PBS3*), and cytokinins (*ARR15*). Previous research studies have showed that plant hormones play a critical role in controlling flowering. Many of these genes have also been reported to play roles in regulating the flowering time and growth development in plants. For example, *PBS3* negatively regulates the flowering time in *Arabidopsis* under LD conditions by modulating the expression of *FLC* and *FLT* [46]. In *Arabidopsis*, *RGL1* is a negative regulator of GA, and *rgl1* mutant exhibits delayed flowering phenotypes [47]. Mutants with functional loss of *IAA7*/*AXR2* flower later under short-day conditions. However, the late-flowering phenotype of these mutants can be rescued by exogenous application of GAs [48]. Therefore, we deduce that overexpression of *GhSWEET42* may influence the expressions of hormone-associated genes, inducing the early-flowering phenotype. TFs play important roles in flowering time regulation [49,50]. Consequently, the TF expression levels in WT and transgenic *Arabidopsis* plants were analyzed, revealing 195 TF-encoding genes with significant expression differences, including 28 WRKY TFs, 26 NAC TFs, 25 ERF TFs, 18 MYB TFs, and 18 bHLH TFs (Table S4). Among these TFs, *WRKY71*, *TGA7*, *NAC2* and *BEE1* are known regulators of the flowering time [4,51,52,53]. Extensive research has demonstrated the important role of *WRKY* genes in regulating the flowering time [51,54]. Among them, *WRKY71* is upregulated in *GhSWEET42*-OE plants. *WRKY71* has been reported to promote early flowering in *Arabidopsis* by activating the expression of *FT* and *LFY* [51]. In *GhSWEET42*-OE plants, the expressions of *TGA7* and *NAC2* are significantly upregulated. *TGA7*, a member of the bZIP TF family, is regulated by autonomous pathway genes and can promote flowering in *Arabidopsis* [52]. *NAC2* has also been reported to delay flowering in *Picea wilsonii* by altering the expression of *FT*, *SOC1*, and *FLC* [53]. In *GhSWEET42*-OE plants, the expression level of the bHLH gene *BEE1* is significantly downregulated. *BEE1* is involved in the photoperiod pathway in *Arabidopsis* and activates the transcription of *FT* to promote flowering [4]. These findings suggest that *GhSWEET42* regulates flowering mainly by altering the expression levels of the TFs *WRKY71* and *TGA7*. Further studies focusing on the downstream target TFs will enhance our understanding of the molecular mechanisms by which *GhSWEET42* influences the flowering time.

## 4. Materials and Methods

### 4.1. Plant Materials and Growth Conditions

For the expression pattern analysis of *GhSWEET42*, two early-maturing cotton (*Gossypium hirsutum* L.) varieties, CCRI50 and CRRI74, along with two non-early-maturing varieties, TM-1 and Lu28, were utilized. These varieties were cultivated in a greenhouse at Liaocheng University, Liaocheng City, Shandong Province (longitude: 115.97° E, latitude: 36.45° N). *Arabidopsis thaliana* plants were grown under two conditions: one chamber with a 16 h light/8 h dark cycle at 25 °C, and another with a 10 h light/14 h dark cycle at 25 °C. Cotton plants were also maintained under a 16 h light/8 h dark cycle at 25 °C.

Leaf blades from CCRI50, CRRI74, Lu28, and TM-1 were sampled at the 2-, 3-, 4-, and 5-leaf stages to analyze the *GhSWEET42* expression patterns across different growth stages. For the tissue-specific expression analysis, roots, stems, leaves, buds, flower buds, petals, and sepals were harvested from CCRI50. Each sample included three biological replicates. All the samples were immediately frozen in liquid nitrogen and stored at −80 °C for the subsequent total RNA extraction.

### 4.2. RNA Extraction and qRT-Time PCR Analysis

RNA was extracted from frozen samples utilizing the FastPure Universal Plant Total RNA Isolation Kit (Nanjing Vazyme Biotech Co., Ltd., Nanjing, China) according to the manufacturer’s protocol. cDNAs synthesis was performed from 2 μg of total RNA using the All-in-One First-Strand Synthesis MasterMix (with dsDNase) from LABLEAD. The relative gene expression levels were quantified via qRT-PCR using the 7500 Real-Time PCR system (Applied Biosystems, Carlsbad, CA, USA) and SYBR Premix Ex TaqTMII (No. DRR041A; TaKaRa Bio Inc., Kusatsu, Shiga, Japan). The endogenous controls employed were *G. hirsutum actin* (*GhActin*) and *A. thaliana UBQ10* (*AtUBQ10*), and the relative expression levels were determined using the 2^−ΔΔCT^ method (Schmittgen TD and Livak KJ, 2008). All the reactions included three biological replicates. The primers for the qRT-PCR were designed using GenScript (https://www.genscript.com/ssl-bin/app/primer, accessed on 13 October 2023).

### 4.3. Gene Cloning and Sequence Analysis

The genomic sequence and CDS of *GhSWEET42* (*GH_ D12G2595*) were retrieved from the National Center for Biotechnology Information (NCBI; https://www.ncbi.nlm.nih.gov/, accessed on 10 March 2023) [35]. The primers were designed with Primer 3 (https://github.com/primer3-org, accessed on 6 April 2023). The CDS of *GhSWEET42* was amplified from TM-1 cDNA as a template. The PCR products were subsequently inserted into the pCAMBIA1305.1 vector for sequencing. The intron–exon structure of the *GhSWEET42* gene was delineated using GSDS2.0 (http://gsds.cbi.pku.edu.cn/, accessed on 12 July 2023). The conserved structural domains of the GhSWEET42 protein were analyzed via SMART (https://smart.embl.de/, accessed on 13 July 2023). Homologs of GhSWEET42 were identified using the NCBI Blastp search program (http://www.ncbi.nlm.nih.gov/, accessed on 25 July 2023).

### 4.4. Sequence Alignment and Phylogenetic Analysis

The multiple sequence alignment was performed with DNAMAN v6.0. A phylogenetic tree was constructed using the maximum likelihood (ML) method in MEGA X [55].

### 4.5. Subcellular Localization Analysis

The *GhSWEET42* coding region, excluding the stop codon, was amplified and fused to the N-terminus of GFP in the pCAMBIA1305.1 vector under the CaMV 35S promoter. The expression vectors carrying the fused protein and the plasma membrane marker *NAA60* were co-transformed into tobacco (*Nicotiana benthamiana*) leaves as previously described [25]. The fluorescence signals were detected using a laser confocal scanning microscope (ZEISS Microsystems LSM 700, Oberkochen, Baden-Württemberg, Germany).

### 4.6. Genetic Transformation of Arabidopsis

The recombinant pCAMBIA1305.1-SWEET42 plasmid was introduced into *Agrobacterium tumefaciens* strain GV3101 cells via chemical transformation. The *Arabidopsis* plants were transformed using the floral dip method [56]. Flower buds of the WT plants were immersed in an Agrobacterium suspension (OD600 = 0.8–1.0) for 30 s and incubated in the dark for 24 h. The transgenic seedlings were screened on solid 1/2 MS agar medium containing hygromycin (25 mg/L). The phenotypic observations and data analyses were conducted in the T3 generation, with rosette leaves collected for the expression analysis.

### 4.7. Measurements of Plant Developmental Traits

The flowering time was quantified as the number of days from germination until the macroscopic visualization of flower buds (1–2 mm) by the naked eye. The number of rosette leaves per plant was counted at the first bloom.

### 4.8. Library Construction and RNA-Seq

The total RNA was extracted from the rosette leaves of the WT and transgenic *Arabidopsis* plants using the FastPure Universal Plant Total RNA Isolation Kit (Nanjing Vazyme Biotech Co., Ltd., Nanjing, China). The concentration and quality of the RNA were assessed using a NanoDrop spectrophotometer (Thermo Scientific, Waltham, MA, USA). Three micrograms of RNA were utilized for the mRNA sample preparation. Sequencing libraries were constructed through the following protocol: mRNA was purified from the total RNA using poly-T oligo-attached magnetic beads. Fragmentation was induced using divalent cations at elevated temperatures in an Illumina proprietary fragmentation buffer. First-strand cDNA synthesis was performed with random oligonucleotides and Super Script II, followed by second-strand cDNA synthesis, which was subsequently performed using DNA Polymerase I and RNase H. The remaining overhangs were converted into blunt ends via exonuclease/polymerase activities, and the enzymes were subsequently removed. Adenylation of the 3′ ends of the DNA fragments was conducted, and Illumina PE adapter oligonucleotides were ligated for hybridization. cDNA fragments of 400–500 bp were purified using the AMPure XP system (Beckman Coulter, Beverly, CA, USA). DNA fragments with ligated adaptor sequences on both ends were selectively enriched using Illumina PCR Primer Cocktail in a 15-cycle PCR reaction. The products were purified using the AMPure XP system and quantified with Agilent’s high-sensitivity DNA assay on a Bioanalyzer 2100 system (Agilent Technologies Inc., Santa Clara, CA, USA). The sequencing library was then sequenced on the NovaSeq 6000 platform (Illumina, San Diego, CA, USA) by Shanghai Personal Biotechnology Co., Ltd., Shanghai, China.

### 4.9. Data Analysis

GraphPad Prism 8.0 (GraphPad Software, La Jolla, CA, USA) facilitated the statistical analyses, employing Student’s *t*-test to assess significance, with the thresholds set at *p* < 0.05 and *p* < 0.01. Sequencing data were filtered using fastp (0.22.0) to generate high-quality sequences (Clean Data) for further analysis. The filtered reads were aligned to the *Arabidopsis* reference genome (https://www.Arabidopsis.org) using HISAT2 (v2.1.0). The differential gene expression was assessed via DESeq (v1.38.3) with the following criteria: |log2FoldChange| > 1 and *p*-value < 0.05. Additionally, bidirectional clustering of all the differentially expressed genes (DEGs) was performed using the R package Pheatmap (v1.0.12), generating a heat map based on the gene expression differences. The Euclidean method was used to calculate the gene distance and clustering. Enrichment analysis of the KEGG pathways for the DEGs was conducted using ClusterProfiler (v4.6.0), focusing on the pathways with a *p*-value < 0.05. The transcription factors and their families were predicted using the PlantTFDB (plant transcription factor database) database, followed by statistical analysis of the predicted transcription factor DEGs.

### 4.10. Primers

The primers utilized in this study are shown in Table S5.

## 5. Conclusions

This research elucidates the functional role of cotton *GhSWEET42* in flowering regulation. Various aspects of the *GhSWEET42* gene were examined, including its subcellular localization and expression profiles across different tissues and developmental stages. Overexpression of *GhSWEET42* in *Arabidopsis* induced an early-flowering phenotype under long-day conditions. RNA-seq analysis revealed significant enrichment of the GO and KEGG pathways and differential expression of the TFs in *GhSWEET42*-OE plants compared to the wild type. This suggests that *GhSWEET42* integrates multiple pathways to modulate the flowering time. This study enhances understanding of *GhSWEET42*’s function and provides valuable insights into the molecular mechanisms governing flowering regulation in cotton.

## Figures and Tables

**Table 1:** Summary of six individually pooled RNA sequencing read counts using *Arabidopsis thaliana* L. as the reference genome.

Sample ID	Raw Read	Clean Reads	Uniquely Mapped Reads	Raw Bases (bp)	Clean Data (bp)	GC (%)	Q20 (%)	Q30 (%)
WT_1	37,702,656	37,354,042 (99.08%)	36,272,152 (97.91%)	5,693,101,056	5,631,084,458	46.14	98.22	94.80
WT_2	38,470,896	38,130,386 (99.11%)	36,921,805 (97.62%)	5,809,105,296	5,748,826,807	45.66	98.30	95.03
WT_3	43,861,112	43,506,840 (99.19%)	42,222,772 (97.72%)	6,623,027,912	6,558,390,861	45.89	97.97	93.97
OE_1	43,389,922	43,041,522 (99.20%)	41,191,592 (97.92%)	6,551,878,222	6,487,103,707	45.46	97.89	93.71
OE_2	64,124,202	63,674,690 (99.30%)	58,737,275 (97.86%)	9,682,754,502	9,597,496,345	45.22	98.08	94.22
OE_3	41,961,324	41,625,310 (99.19%)	40,246,839 (98.03%)	6,336,159,924	6,275,413,217	45.87	98.47	95.47

## Data Availability

Data are contained within the article and Supplementary Materials.

## Supplementary Materials

The following supporting information can be downloaded at https://www.mdpi.com/article/10.3390/plants13162181/s1, Figure S1: Sequence characteristics of GhSWEET42. (A) Domain structure of GhSWEET42. Amino acids are indicated by blue lines, and transmembrane regions are indicated by red lines. (B) Multiple sequence alignment analysis of the GhSWEET42 protein with its homologs in other species. The transmembrane region is indicated by red lines; Figure S2: Overexpression of *GhSWEET42* in *Arabidopsis* under SD conditions. (A) Phenotypes of WT and *GhSWEET42* transgenic lines under SD conditions; and (B) rosette leaf counts in WT and *GhSWEET42* transgenic lines under SD conditions; Figure S3: Differential TF analysis. TFs in blue indicate expression upregulation, and yellow indicates expression downregulation; Table S1: Differentially expressed genes between WT and *GhSWEET42*-OE plants; Table S2: GO enrichment analysis of the DEGs between WT and *GhSWEET42*-OE plants; Table S3: KEGG enrichment analysis of the DEGs between WT and *GhSWEET42*-OE plants; Table S4: Distribution of the differentially expressed TFs; Table S5: Primer sequences used in this study.
