# Supplementary figures and images for: GhSWEET42 Regulates Flowering Time under Long-Day Conditions in Arabidopsis thaliana
Source: Plants (Basel). 2024 Aug 6;13(16):2181. doi: 10.3390/plants13162181 (PMC11360393; doi:10.3390/plants13162181)

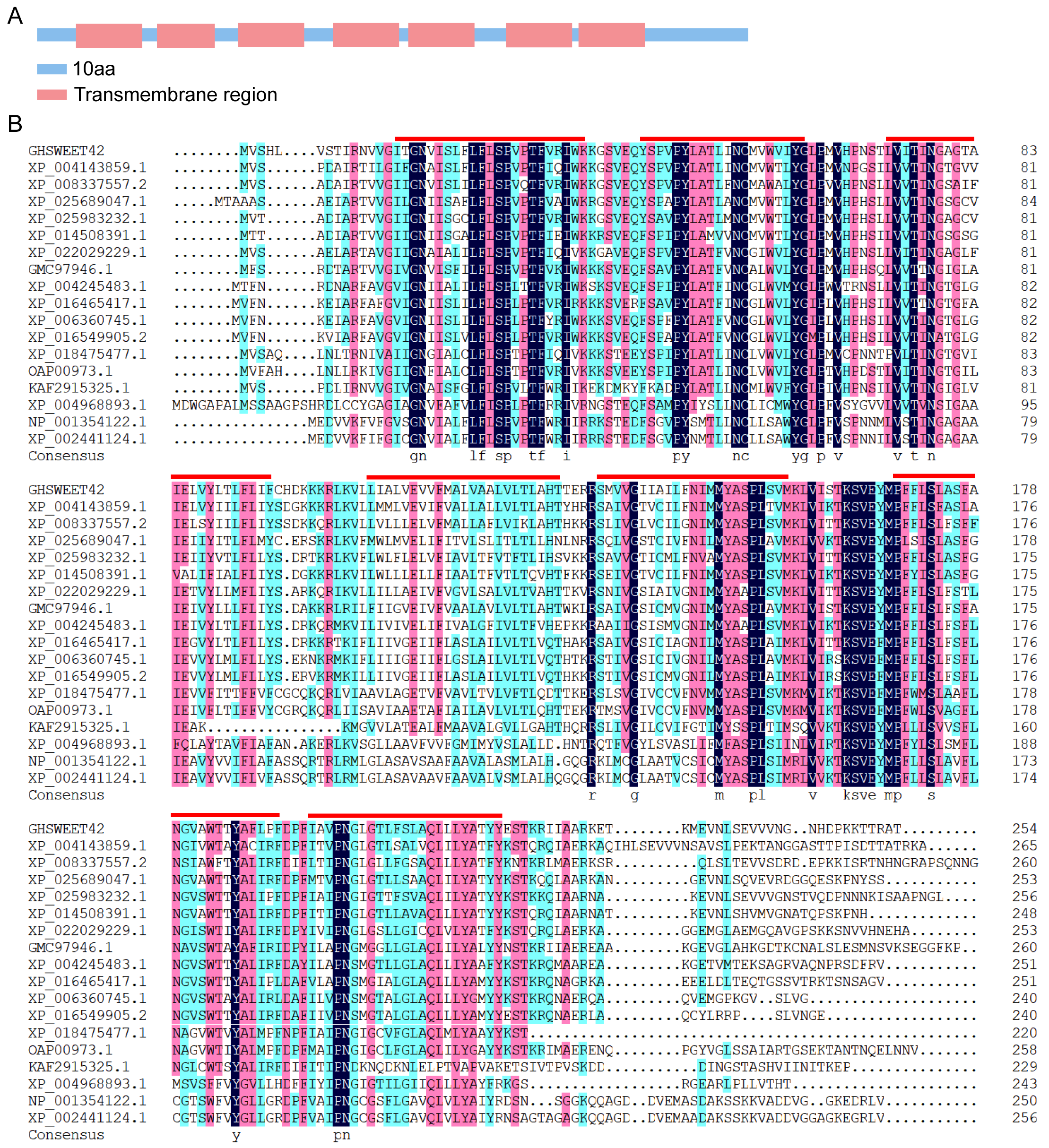

Supplement: Supplementary file 1 [file plants-13-02181-s001.zip › Figure S1.tif]

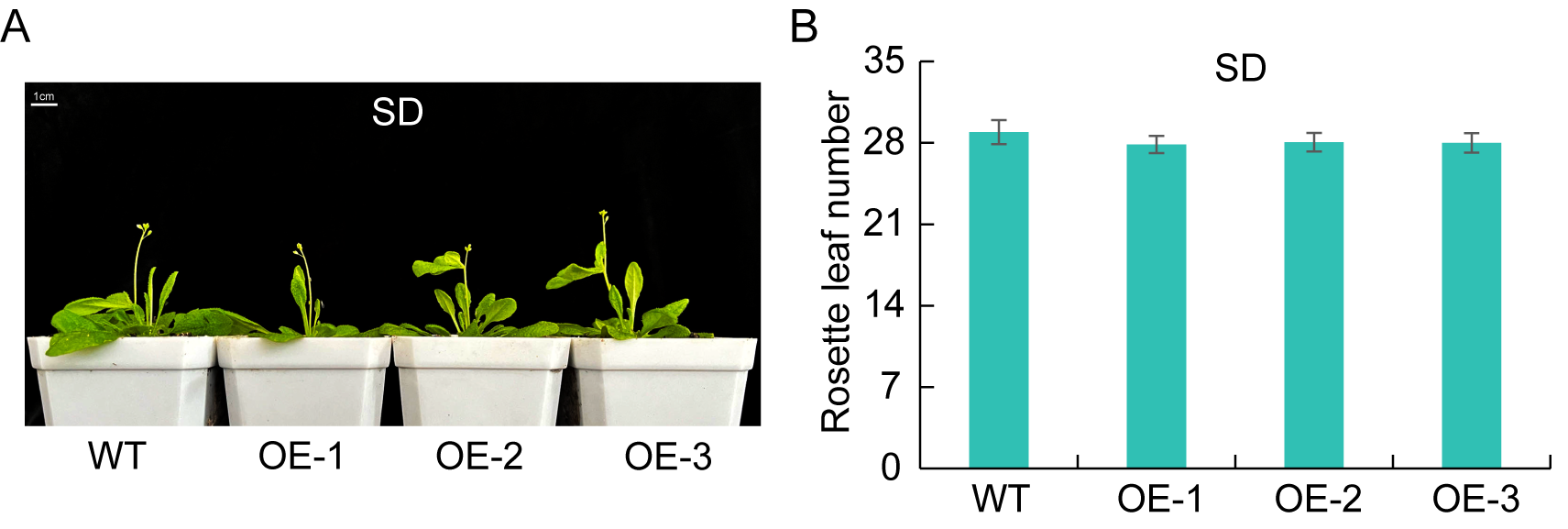

Supplement: Supplementary file 1 [file plants-13-02181-s001.zip › Figure S2.tif]

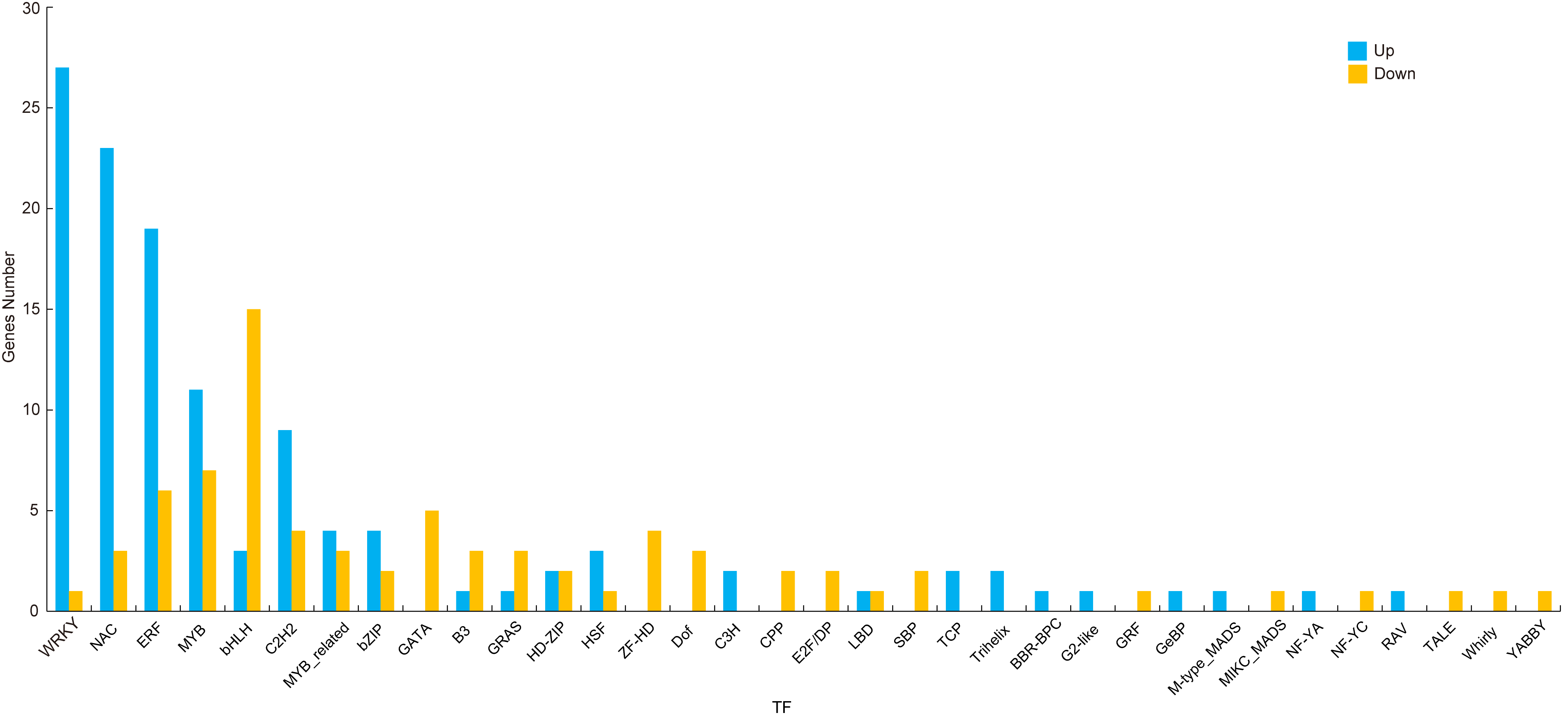

Supplement: Supplementary file 1 [file plants-13-02181-s001.zip › Figure S3.tif]
